# Composition of weathering crusts on sandstones from natural outcrops and architectonic elements in an urban environment

**DOI:** 10.1007/s11356-014-3312-y

**Published:** 2014-07-20

**Authors:** Mariola Marszałek, Zofia Alexandrowicz, Grzegorz Rzepa

**Affiliations:** 1 Department of Mineralogy, Petrography and Geochemistry, AGH University of Science and Technology, al. A. Mickiewicza 30, 30-059 Kraków, Poland; 2 Institute of Nature Conservation, Polish Academy of Sciences, al. A. Mickiewicza 33, 31-120 Kraków, Poland

**Keywords:** Sandstones, Tors, Monuments, Weathering crust, Air pollution

## Abstract

This work presents mineralogical and chemical characteristics of weathering crusts developed on sandstones exposed to various air pollution conditions. The samples have been collected from sandstone tors in the Carpathian Foothill and from buildings in Kraków. It has been stated that these crusts differ in both fabric and composition. The sandstone black crust from tors is rich in organic matter and composed of amorphous silica. Sulphate incrustations accompanied by dust particles have been only sometimes observed. Beneath the black crust, a zone coloured by iron (oxyhydr)oxides occurs. The enrichment of the surface crust in silica and iron compounds protects the rock interior from atmospheric impact. The sandstones from architectonic details are also covered by a thin carbon-rich black crust, but they are visibly loosened. Numerous salts, mainly gypsum and halite, crystallise here, thus enhancing deterioration of the rock. Moreover, spherical particles originated from industrial emissions are much more common. Gypsum in natural outcrops, forms isolated and well-developed crystals, whilst these found on the architectonic details are finer and densely cover the surface. Such diversity reflects various concentrations of acid air pollutants in solutions.

## Introduction

The course of weathering of exposed rock surfaces depends on both internal factors, such as mineral composition, structure and texture of the rock considered, and external ones, for instance, climate and anthropogenic pressure. The changes affect particularly the porous rocks whose minerals are relatively less resistant to weathering and lead to the development of surface crusts. For a certain time, the crust may protect the subsurface layer of the rock, but it can also be destructive, boosting exfoliation of the rock surface. The impact of anthropogenic factors, particularly of air pollution, increases the intensity of weathering and the formation of secondary minerals. These are primarily salts that form black crust and efflorescences on the rock surfaces and accumulate in subsurface layers (Thomachot and Jeannette 2004; Charola et al. 2006). Such changes affect various rock types, as limestones (Bede 2000; Marszałek 2000; Belafiore et al. 2013), granitoids (Labus 1998; Schiavon 2000), sandstones (Bai et al. 2003) and also masonry walls (Gentilini et al. 2012). The mechanism and path of these processes have been well known and explained (e.g. Goudie and Viles 1997; Charola 2000; Doehne 2002; Espinoza-Marzal and Scherer 2010; Adamovič et al. 2011; Ludovico-Marques and Chastre 2012; Navrátil et al. 2013). In the case of sandstones, the salt weathering usually leads to their disintegration due to an increase in intergranular pressure caused by growing salt crystals, hydration of salts (Goudie and Viles 1997; Winkler 1997) and dissolution of silicates by saline fluids. On the other hand, in fine-grained polymictic sandstones rich in clay minerals, case hardening may occur as well (Adamovič et al. 2011). Only few studies, however, focused on comparing the structures and compositions of weathering crusts developing in various environments but on comparable rock substrates (Siedel and Klemm 2001; Bai et al. 2003; Wilczyńska-Michalik 2004; Přikryl 2007). The present authors investigated secondary substances of crusts formed on sandstones influenced to various degrees by air pollutants. The aims of this paper are to study the damage mechanisms affecting lithologically and genetically similar sandstones in the natural outcrops and in the urban environment and to correlate the patterns and composition of the weathering crust with different components of atmospheric aerosol.

**Fig. 1:**
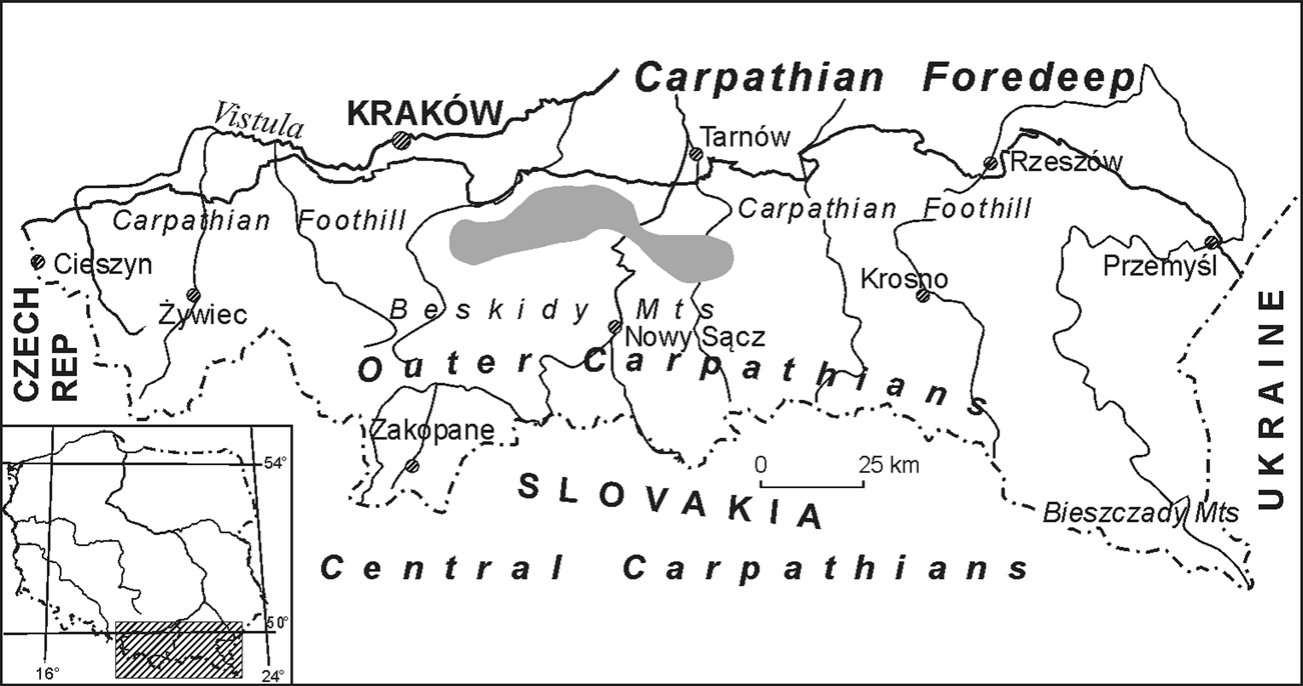
Sampling sites of the weathering crusts developed on sandstone surfaces of architectonic details in Kraków and on natural tors in the Carpathian Foothill (the latter marked in *grey*)

The analyses were conducted on the outermost parts of weathering crusts, black or dark grey in colour, developed on the surfaces of natural sandstone tors occurring within the Carpathian Foothill of the Polish Outer (Flysch) Carpathians (Fig. 1), and the crusts that grow on analogous sandstones used in historic buildings within the urban area of Kraków, a town placed on the UNESCO World Heritage List. Unfortunately, Kraków is one of the European towns with the most polluted atmosphere (e.g. Fenger 1999; Mira-Salama et al. 2008; Juda-Rezler et al. 2011).

**Fig. 2:**
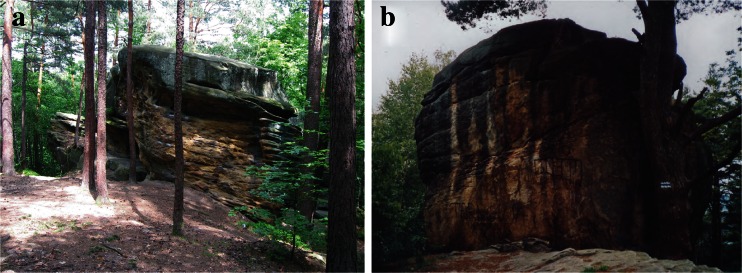
**a** The Brodziński Stones Natural Monument in the Carpathian Foothill. Thick bedding of the massive sandstone facilitated the exploitation. **b** Sandstone tower rock in the Stone Town Nature Reserve in the Carpathian Foothill. The exposed surfaces of the rock are covered with black crusts. Photos by Z. Alexandrowicz

The Outer Carpathians, whose age spans Upper Jurassic and Neogene, are composed mainly of flysch, i.e. alternating complexes of sandstones, mudstones and shales (Oszczypko 2004, 2006; Poprawa and Malata 2006). Among dominating thin- and medium-layered strata are widely developed lithofacies of thick-bedded sandstones and conglomerates. Many outcrops of these rocks were shaped during Quaternary into tors due to various denudation processes. Particularly prone to such changes in the Carpathian Foothill are the complexes of the Istebna sandstones (Upper Cretaceous–Lower Eocene) and the Ciężkowice sandstones (Lower Eocene), which reveal similar lithological and sedimentary features, typical of submarine flows (fluxoturbidites) (Alexandrowicz 1978; Leszczyński 1981; Oszczypko 2004). These sandstones are usually coarse-grained, in places conglomeratic, poorly sorted, developed as quartz-rich oligomictites, but also arkoses and greywackes (Kamieński et al. 1967). Their cement is scarce, composed of clay minerals, sometimes of silica, occasionally of calcite. The sandstone complexes mentioned consist of very thick beds (often above several meters) and are characterised by high water absorbability, low or medium compression strength and very high frost resistance (Bromowicz et al. 1976). This lithological type of sandstones has been quarried within the Carpathian Foothill since the early Middle Ages. The Carpathian sandstones have been used in constructing works, as the rocks perfectly suitable for building purposes because of their good physical and chemical parameters. Other reasons are of the logistic nature (Rajchel 2004, 2008; Bromowicz 2009; Bromowicz and Magiera 2010, 2013): the stones were quarried chiefly within the Carpathian Foothill, just in a short distance south of Kraków (Fig. 1), from where could be transported in large blocks (Fig. 2a). Therefore, they were widely used in Kraków in the Romanesque, Gothic, Renaissance and nineteenth-century constructions (Rajchel 2004).

Current quarrying of these sandstones is limited. Of around 1,000 quarries of various sandstones that have been recorded in the Carpathians (Rajchel 2004), currently active are 36 operations (Brzeziński 2013). On the other hand, some of the quarries and most of natural exposures of sandstone tors are subject of protection as examples of various types of sandstones, elements of the natural landscape, and also due to the presence of their specific sedimentary structures and weathering processes (Alexandrowicz 2008, 2009). They have been protected separately as nine reserves and 38 monuments of the inanimate nature (Fig. 2a, b), and additionally, a number of such tors are situated within national parks and landscape parks (Alexandrowicz and Poprawa 2000).

## Materials

The crust samples occurring in their natural environment were obtained from the tors comprised the Istebna and Ciężkowice sandstones of the Silesian Unit (Upper Cretaceous–Lower Eocene) of the Outer (Flysch) Carpathians. In terms of lithology, the sandstones considered are similar. Their surface is covered by a weathering crust, usually 1–2-cm thick (it can reach a maximum of 10 cm), characterised by lamination, either continuous or developed in patches, with zones of variable colour and thickness running parallel to the rock surface (Alexandrowicz and Pawlikowski 1982; Rzepa et al. 2011; Alexandrowicz et al. 2012; Marszałek et al. 2012). The lamination is chiefly associated with variable proportions and mineralogy of iron compounds and is a separate issue discussed in detail by Alexandrowicz et al. (2012, 2014) and Rzepa et al. (2011). The outermost part of the weathering crust is often developed as a thin hard, black or dark grey layer. Because of its properties, chiefly the hardness associated with the presence of, e.g. amorphous silica, it closely resembles the *varnish* type covers described primarily on rocks exposed in deserts and only sometimes on the surfaces of sandstone tors in Europe (Dorn 1984; Robinson and Williams 1986; Cílek 1997; Thiry 2005; Dorn and Krinsley 2011). It is also termed *patina*, whereas its analogues formed as a result of pollution on stony architectonic elements in urban areas are called *false patinas* (Manecki et al. 1982, 1997; Labus 2008). The present authors have focused on the external, black or grey layers of sandstone weathering crusts from sites with rocky forms occurring within forest enclaves in the Carpathian Foothill: the Brodziński Stones, the Stone Town Nature Reserve, the Mushroom Stone, Kobyla Góra and Szczyżyc. A total of 30 samples were collected for the study, and the results of investigating 14 of them have been presented here. The weathering crusts on natural rocks have mostly the area not exceeding 25 cm^2^ and are several centimetres thick. The samples selected reveal a distinctly developed black or dark grey outer layer affected by atmospheric factors and are usually clearly laminated underneath.

**Fig. 3:**
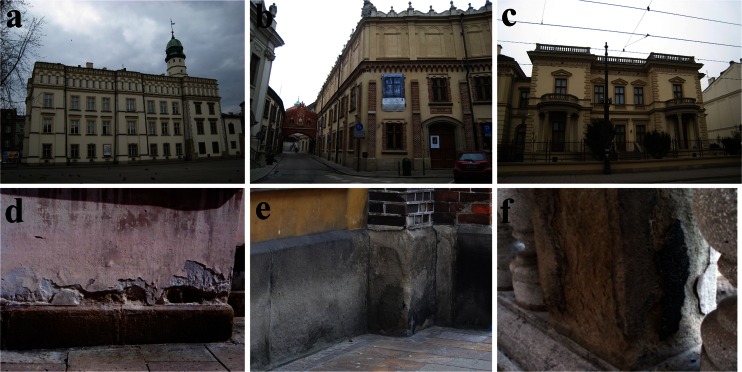
General view and examples of black crust development of: **a**, **d** the Ethnographic Museum, **b**, **e** the Czartoryski Museum and **c**, **f** the Czapski Palace. Photos: **d** by T. Sokal, **e** by A. Bohan-Strumińska, **f **by A. Orzechowska

The samples of grey and black weathering crusts on sandstone architectonic elements were collected from three historical buildings situated near the centre of Kraków: the Ethnographic Museum, the Czartoryski Museum and the Czapski Palace (Fig. 3).

The history of the first of the buildings, which has been housing the Ethnographic Museum since 1947, dates back to 1414, when construction of the City Hall began in Kazimierz, a satellite town of Kraków into which it was incorporated at the beginning of the nineteenth century. In the last two centuries, the building has been repeatedly rebuilt and extended, assuming its recent shape in 1962–1966 as a result of a major restoration (Rożek 2000).

The Czartoryski Museum (a branch of the National Museum) is the oldest institution of this type in Poland, established in 1801 in Puławy (central Poland) by Princess Izabela Czartoryska (née Flemming), who belonged to an eminent Polish aristocratic family (Rożek 2000). Transferred to Kraków in the mid-nineteenth century, the collection was placed in building of the Municipal Arsenal, remodelled in the same time into a small castle-mansion in the early-Renaissance style. The collection was also housed in neighbouring buildings: part of an old Piarist monastery and three tenement houses that were merged in the nineteenth century into a single Czartoryski Palace building. The Arsenal, or so-called the Small Monastery, and the Palace are linked by corridor-like, overhead passages. The single most valuable piece of art in the museum is the *Lady with an Ermine* by Leonardo da Vinci.

The Czapski Palace (also a branch of the National Museum) was erected in 1884. Its owner, Emeryk Hutten-Czapski, placed his valuable numismatic collection there, which he donated next to the city of Kraków and its National Museum (Rożek 2000). At present, the building houses the European Centre of Polish Numismatics.

The samples of sandstone weathering crusts from the Ethnographic Museum (three specimens) were collected from the portal of the main entrance to the building and from the plinth (at a height of up to 1 m above the ground). The samples from the Czartoryski Museum (seven specimens) were collected from plinths and window sills at a similar height, whereas those from the Czapski Palace (four specimens) from columns of a balcony. The samples were collected during the dry season, in June and July of 2011, utilizing a restoration campaign.

Although the study was to be centred on the outermost sandstone layers, i.e. black crusts, also the altered sandstone zones located directly below were investigated as the so-called weathering zones. The black crust and the weathering zone are collectively termed the weathering crust.

Depending on the local conditions, samples were collected with a hammer or a knife; their volume ranged from single to several tens cubic centimetres. Sample descriptions and localisations are given in Table 1.

**Table 1:** List of samples, sampling sites and methods of analysis

Sample location		Samples	Site and height above the ground (m)	Analysis scope				
				OM	SEM	XRD	TA	Chemical analysis
Carpathian Foothill	The Mushroom Stone	KG-1	Sandstone tor; 1.2	+	+	+		
	Kobyla Góra	CH-1	Sandstone tor; 1	+	+	+		
	The Brodziński Stones	KB-1	Sandstone tor; 1.5	+	+	+		
		KB-2	Sandstone tor; 2.5	+	+	+	+	
		KB-6	Sandstone tor; 1.7	+	+			
		KB-8	Sandstone tor; 1.5	+	+			
		KB-9	Sandstone tor; 1	+	+	+		
		KB-10	Sandstone tor; 1	+	+	+	+	+
	Szczyżyc	SZ-1	Sandstone tor; 1.3	+	+			
	The Stone Town Nature Reserve	SMC-1	Sandstone tor; 1.5	+	+	+		
		SMC-11	Sandstone tor; 1	+	+	+	+	+
		SMC-12	Sandstone tor; 1.5	+	+	+		
		SMC-13	Sandstone tor; 1	+	+	+	+	+
		SMC-14	Sandstone tor; 1.5	+	+	+	+	+
Kraków	The Ethnographic Museum	Me-81	Portal fragment; 0.6	+	+	+	+	+
		Me-82	Plinth fragment; 0.3	+	+	+		
		Me-83	Plinth fragment; 0.1	+	+	+	+	
	The Czartoryski Museum	MC-001	Plinth fragment; 0.7	+	+			
		MC-008	Plinth fragment; 0.5		+	+		
		MC-009	Plinth fragment; 0.7	+	+	+	+	
		MC-010	Plinth fragment; 1.3		+			
		MC-011	Window still fragment; 1.7		+	+		
		MC-012	Window still fragment; 1.7		+	+		
		MC-013	Window still fragment; 1.7		+			
	The Czapski Palace	PC-01	Balcony column; 0.3	+	+	+	+	+
		PC-03	Balcony column; 0.4		+	+		
		PC-04	Balcony column; 0.6	+	+	+		
		PC-05	Balcony column; 0.8		+			

*OM* optical microscopy, *SEM* SEM-EDS, *TA* thermal analysis (TG/DTA), *+*indicate that the analyses have been done

## Methods

Microscopic observations (including petrographic descriptions of natural unaltered rocks as well) were carried out using optical transmission microscopy with an Olympus BX-51 instrument and an Olympus DP-12 digital camera. Detailed studies of the components of weathered crusts were performed by the scanning electron microscopy (SEM), using a FEI 200 Quanta FEG microscope with an EDS/EDAX spectrometer. The excitation voltage was 20 kV and the pressure 60 Pa (low vacuum, the samples were not coated).

The phase composition was determined using X-ray diffractometry (XRD) with a Philips APD PW 3020 X’Pert instrument. The samples were ground in an agate mortar. The measurement conditions were as follows: anode Cu_Kα_, generator settings 35 kV and 30 mA, recording range 3–70°2*θ*, step size 0.05°, counting time 1 s per step. The XRD patterns were evaluated by an XRAYAN software using a diffraction pattern database of International Centre for Diffraction Data (The Powder Diffraction File PDF-4^+^ 2013).

The thermal analyses (thermogravimetry and differential thermal analysis -TG/DTA) were conducted with a Derivatograph C apparatus made by MOM Budapest. The powdered sample with a weight of about 100 mg was heated in the air in the range 20–1,000 °C with the constant heating rate of 10 °C/min. Al_2_O_3_ powder was used as the thermally inert substance.

The chemical compositions were determined by applying different analytical methods at the Activation Laboratories Ltd. (ACTLABS) in Canada. Concentrations of major compounds (SiO_2_, Al_2_O_3_, Fe_2_O_3_, MnO, MgO, CaO, Na_2_O, K_2_O, TiO_2_ and P_2_O_5_)_._ were measured using inductively coupled plasma optical emission spectrometry (ICP-OES) method after fusion of the ground sample with a mixture of lithium metaborate and lithium tetraborate in an induction furnace followed by dissolution of the melt in 5 % nitric acid. Trace element concentrations were determined either with inductively coupled plasma mass spectrometry (ICP-MS) (Be, Bi, Cd, Cu, Mo, Ni, Pb, Sr, V, Y, Zn, Zr) or with instrumental neutron activation analysis (INAA) (Au, Ag, As, Ba, Br, Co, Cr, Cs, Hf, Hg, Ir, Rb, REE, Sb, Sc, Se, Ta, Th, U, W). The samples for ICP-MS analyses were prepared by the digestion of a material with a mixture of concentrated HClO_4_, HNO_3_, HCl and HF at 200 °C to fuming and then dilution with aqua regia. INAA analyses were carried out as follows (Hoffmann 1992): a 1 g aliquot of the sample was encapsulated in a polyethylene vial and irradiated with flux wires and an internal standard at a thermal neutron flux of 7 × 10^12^ n cm^−2^ s^−1^. After a 7-day period, the samples were counted on a high purity Ge detector with resolution of better than 1.7 keV for the 1,332 keV Co-60 photo peak. Using the flux wires, the decay-corrected activities were compared to a calibration developed from multiple certified international reference materials.

Optical and scanning electron observations were made on universal polished thin sections cut perpendicular to stone surfaces. Additionally, broken surfaces of outer parts were studied using the SEM method. The phase and chemical composition were established for samples of outermost parts of the sandstones (layer reaching to ca. 0.5 cm from the surface) and inner part (ca. 2 cm below the surface).

Due to a limited size of samples, it was impossible to conduct all determinations for each of them. Therefore, full range of analyses was carried out only for selected, representative samples (Table 1).

## Results and discussion

**Fig. 4:**
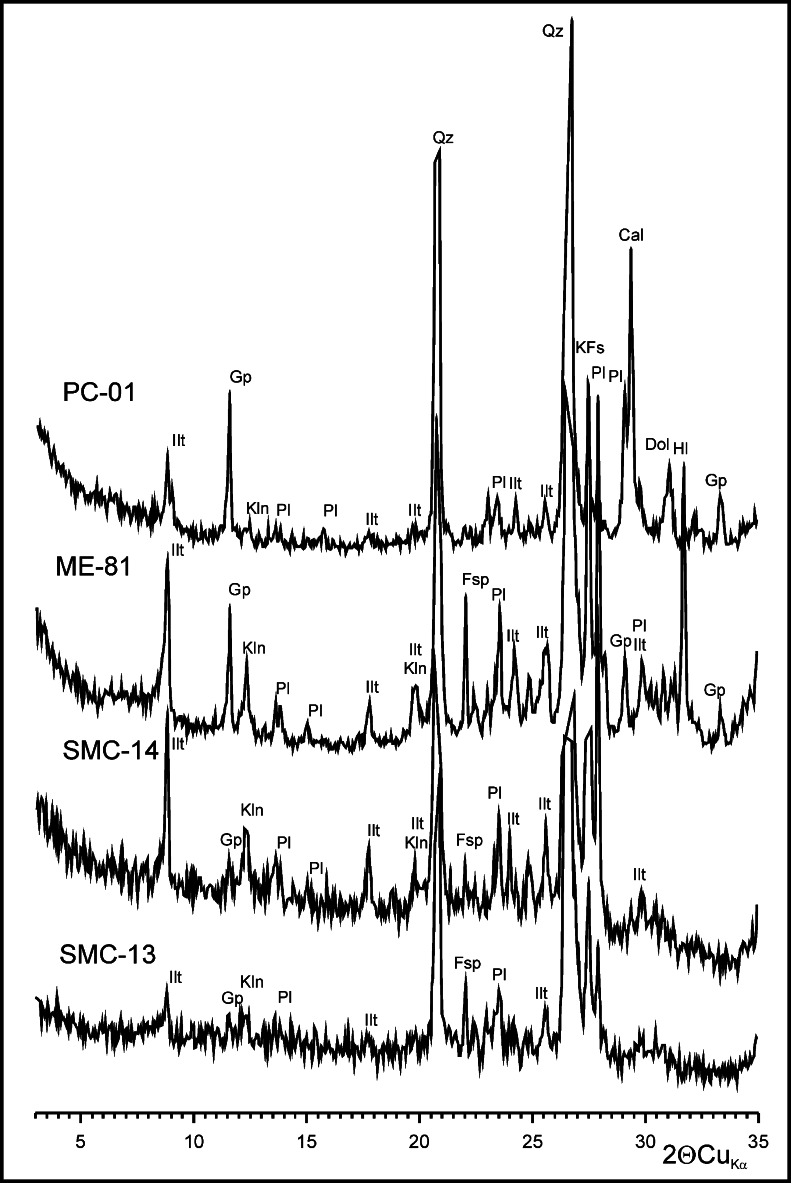
XRD patterns—weathering crusts from natural outcrops (SMC-13, SMC-14) and architectonic details of the Ethnographic Museum (ME-81) and the Czapski Palace (PC-01). *Ilt* illite, *Kln* kaolinite, *Pl* plagioclase, *Gp* gypsum, *Fsp* feldspar, *KFs* K-feldspar, *Qz* quartz, *Cal* calcite, *Dol* dolomite, *Hl* halite

All the rocks examined are medium-grained sandstones with very similar lithological characteristics. Their grain framework comprises quartz, feldspars, rock fragments, micas (biotite and muscovite) and accessory minerals (e.g. rutile, ilmenite, zircon, tourmaline and opaques, including pyrite). Feldspars are significantly altered by kaolinitisation and sericitisation. Also altered are the remaining, albeit less resistant aluminosilicates. The contact-porous cement is composed chiefly of clay minerals and iron (oxyhydr)oxides. It is locally enriched in carbonates, while glauconite aggregates were also found in some places. The XRD analysis has revealed among clay components kaolinite, illite and probably illite-smectite mixed-layer minerals (Fig. 4).

### Weathering crusts on sandstone tors

**Fig. 5:**
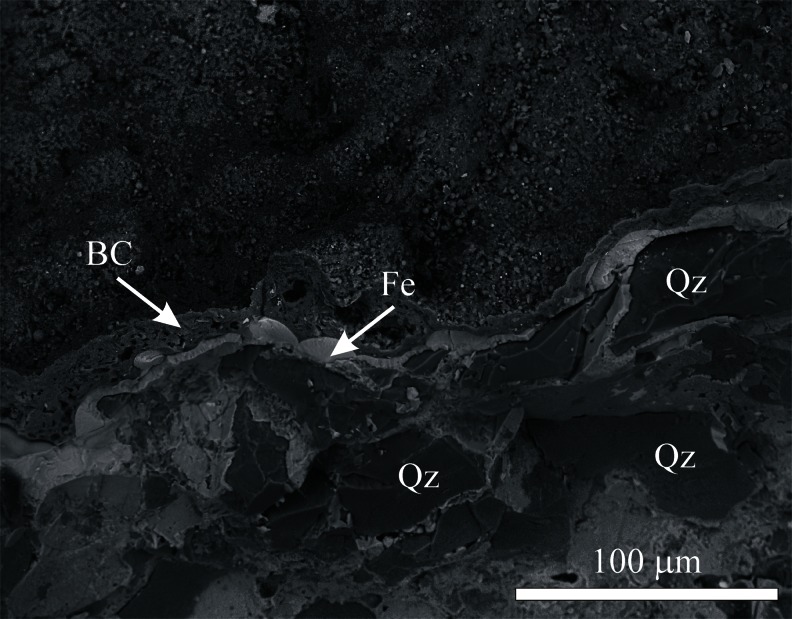
Scanning electron microscope image of the weathering crust developed on the sandstone tor surface (sample KB-9). *BC* black crust, *Fe* iron (oxyhydr)oxides incrustation between quartz grains, *Qz* quartz

**Fig. 6:**
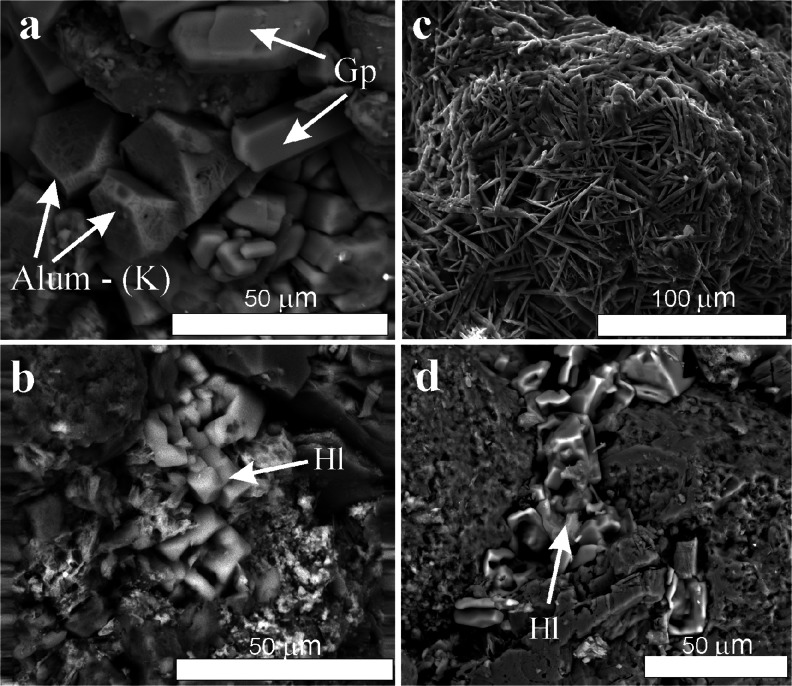
Scanning electron microscope images of the sandstone surface: **a**, **b** from natural outcrops (sample SMC-13) and **c**, **d** from architectonic details (sample ME-81). *Gp* gypsum, *Alum-(K)* potassium alum, *Hl* halite, partial dissolution of mineral grains is also visible (**d**)

The outer, altered rock crusts (a few centimetres thick) are covered by a thin, hard, often discontinuous dark grey to black layer of variable morphology. As the identification of the components of this layer under an optical microscope was not possible because of their cryptocrystalline nature, the electron scanning microscope observations provided more information (Fig. 5). The framework minerals of the outer zone are covered by a thin (several to several dozen micrometers thick), amorphous film of a variable chemical composition, which includes C, Si, Al, Fe, P, Cl and K. Amorphous silica predominates among its components, accompanied by organic matter in various morphological forms, occasionally found grains of iron oxides and spherical particles of aluminosilicate glass from industrial emissions. Of salt minerals, euhedral crystals of gypsum (CaSO_4_ · 2H_2_O), barite (BaSO_4_) and potassium alum [KAl(SO_4_)_2_ · 12H_2_O] were observed (Fig. 6a, b). Gypsum was also found in deeper layers of the crust (up to 1 cm below the surface) in the intergranular spaces and pores, together with clay minerals and iron compounds. Another sulphate, jarosite [KFe_3_(OH)_8_(SO_4_)_2_], sporadically occurs in the intergranular spaces, whereas on the crust surface, single crystals of halite (NaCl) and syngenite [K_2_Ca(SO_4_)_2_ · H_2_O] were found.

**Fig. 7:**
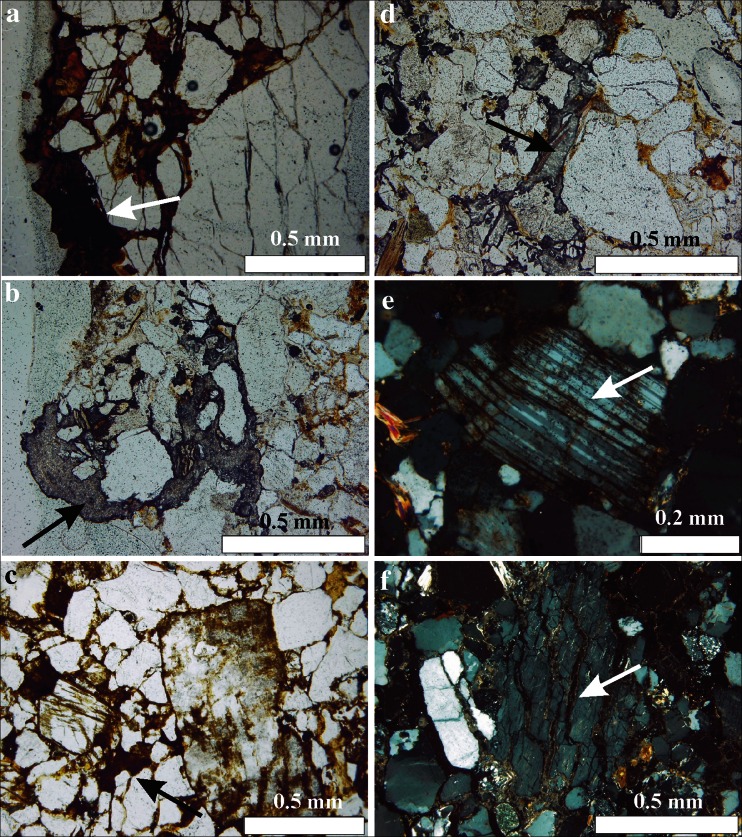
Microphotographs: sandstone from natural outcrops—**a** outer layer covered by ferruginous compounds (sample SMC-14, 1P), **b** iron oxides between mineral grains (sample SMC-12, 1P), and **c** iron compounds occurring in cleavage fissures of feldspar (sample SMC-14, XP); sandstone from architectonic detail—**d** black crust composed of gypsum covering the outer layer of the detail (sample MC-001, 1P), **e** gypsum crystals filling pores and cracks, replacing stone cement (sample MC-001, 1P), and **f** gypsum filling cleavage on feldspar (sample MC-011, XP). Optical microscope, thin sections cut perpendicular to the stone surface. *1P* one polar, *XP* crossed polars

Immediately beneath the above described hard and black crust, there is a zone of sandstone altered by weathering (Fig. 7a, b), which differs from the inner parts of the rock due to the presence of variably coloured zones running parallel to the surface. Alexandrowicz et al. (2012, 2014) have found that the darker layers contain more iron compounds than the lighter ones, whereas their colour depends on the prevailing type of the Fe-bearing minerals. The red and pink zones contain mostly an oxide–hematite (α-Fe_2_O_3_), whilst the yellow and brown zones are characterised by the presence of an oxyhydroxide–goethite (α-FeOOH). The layers closer to the surface of the weathering crust contain often major hematite and are richer in iron (Fig. 7a), whereas the deeper layers are richer in goethite (Fig. 7b). Such stratification is a result of redistribution of iron during weathering. Iron released during the decomposition of primary aluminosilicates, and—to a lesser extent—of carbonates and sulphides, is transported towards the rock surface, where it precipitates and can undergo further transformations (Alexandrowicz et al. 2012, 2014). Additionally, physical changes of rock-forming minerals have been noted in the weathering crust in the form of cracks in quartz grains and fissures running along the cleavage surfaces of feldspars. These cracks and fissures are filled with iron compounds, principally goethite and hematite, whose particles were also found within other minerals (Fig. 7c).

The XRD analyses confirmed the presence of quartz, feldspars, clay and carbonate minerals (Fig. 4). No salt minerals identified in the SEM-EDS examinations were noted, except for gypsum but only in single samples. It is a proof of minor amounts of the salt-group phases in the overall composition of the crust.

The TG/DTA thermal examinations have been rather inconclusive as they indicated, obviously, major, natural sandstone components: quartz, clay and carbonate minerals. The thermal curves obtained for the surface-closest crust samples point to a higher content of iron oxyhydroxides and a lower content of clay minerals than those of the inner parts of the rock (Alexandrowicz et al. 2014). The thermal effects of salt minerals have not been recorded.

The precipitation of amorphous silica (silicification) leads to a hardening and sealing of the rock surfaces. The hydrolytic decomposition of less resistant aluminosilicate minerals, such as feldspars and biotite, which to a variable degree have been replaced by clay minerals and iron compounds, is the main source of the silica. Dissolution of aluminosilicates is favoured by an acidic reaction within the rock, low pH being a result of weathering of sulphides and precipitating iron (oxyhydr)oxides, as well as intensive microbial activity or infiltration of acid waters from atmospheric precipitation (Mcarthur et al. 1991; Bingham and Nordstrom 2000). The first two processes seem to be more significant in the sandstones studied (Alexandrowicz et al. 2012).

The dark grey to black colour of the outer layer can be associated with the presence of atmospheric dusts of industrial origin and also an activity of microorganisms, such as cyanobacteria, bacteria oxidising Fe and Mn, and melanin-producing fungi and actinomycetes (Gaylarde et al. 2007; Corenblit et al. 2011; Dorn and Krinsley 2011; Dakal and Cameotra 2012).

The presence of salts (e.g. gypsum, barite, potassium alum, jarosite, syngenite and halite) results from evaporation. The majority of anions necessary to form these minerals are probably associated with the effect of air pollutants (e.g. Lentschig-Sommer 1960; Jabłońska et al. 2001; Wilczyńska-Michalik 2004; Přikryl et al. 2007). The decomposition of sulphides (pyrite) present in sandstones is a probable source of sulphate ions (Soukupová et al. 2002; Schweigstillová and Hradil 2007). The last process must be particularly important in the case of jarosite, whose formation requires a highly acidic environment (Bingham and Nordstrom 2000). The alteration of aluminosilicate (e.g. feldspars, micas, glauconite and clay minerals) and carbonate rock components is the chief source of cations: K, Ca, Na, Ba and Fe (e.g. Steiger 2003; Wilczyńska-Michalik 2004). However, a connection of these cations with atmospheric particulate pollutants is also probable (Wilczyńska-Michalik 2004; Kamh 2005; Přikryl et al. 2007; Cnudde et al. 2009). The supply of ions via capillary transport of groundwater is less likely.

### Weathering crusts on architectonic details

The sandstones of the outer zones of architectonic details are generally loose and friable. Their surface is covered by a discontinuous, grey or black crust up to 0.5-mm thick. Optical microscopy has revealed only opaque and/or translucent grains of variable morphology, probably iron (oxyhydr)oxides and carbon particles, and transparent gypsum crystals that are usually very fine. Gypsum also fills the cracks in quartz grains and the fissures along the cleavage surfaces of feldspars, as well as spaces between flakes of micas. In some places, it also replaces rock cement (Fig. 7d–f).

**Fig. 8:**
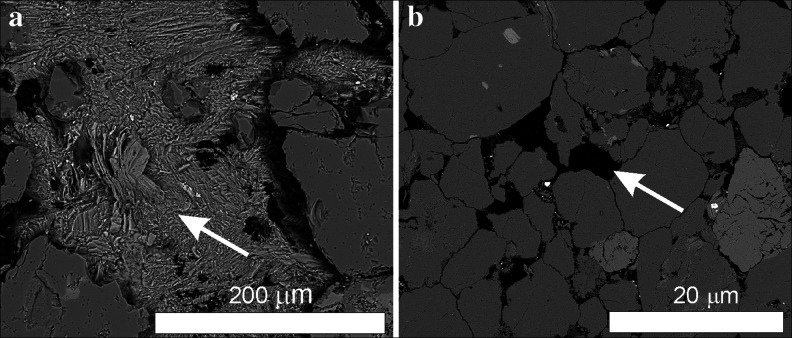
Scanning electron microscope images of sandstone samples from architectonic detail: **a** gypsum replacing cement of the stone (sample MC-010) and **b** empties after leaching the sandstone cement (sample ME-81)

In SEM-EDS examinations, the near-surface framework minerals of sandstones are covered by a thin layer rich in C, Si, Al, Fe, P, Cl, K and Ba. Grain components include iron oxides, along with local occurrences of barite and carbon particles (soot). Above all, however, discontinuous accumulations, containing chiefly salt minerals, mainly gypsum and halite (Fig. 6c, d), spherical particles of aluminosilicate glass and spherules of iron oxides (magnetite and hematite), were observed. The properties and composition of the latter components indicate them to originate from industrial emissions (Marszałek 2008). Another significant component of this layer is organic matter (confirmed in TG/DTA analyses). SEM observations corroborated that numerous laths of gypsum cover the crust surface and also occur within pores of the sandstones, where they replace a primary cement (Fig. 8a). Sometimes, however, the intergranular spaces after leaching the cement remain empty (Fig. 8b) and increase the porosity of the stone. The framework components commonly show traces of chemical corrosion (Fig. 6d). In samples from the Czartoryski Museum and the Ethnographic Museum collected at a small distance from the ground level, halite and gypsum are frequent secondary minerals. They precipitated primarily in the intergranular spaces (Fig. 6d) and in some areas densely cover the surface. Halite is absent, however, in the samples collected from the balcony columns of the Czapski Palace.

The presence of these salt minerals was also confirmed by XRD analyses, which is a proof of their significant presence in the composition of black crust (X-ray detection limit of salt minerals is relatively high, being around 3 %). Gypsum has also been found in deeper layers (ca. 2 cm below the surface).

The dark grey to black colour of the crust surfaces results chiefly from the presence of atmospheric dusts. However, an impact of microorganisms, similar to that on natural outcrops, cannot be excluded (Saiz-Jimenez 2003).

Intensive destruction of sandstones indicates that natural weathering is enhanced by processes of anthropogenic nature. Significant amounts of gypsum and halite are, among others, effects of such pollution and due to easy crystallisation and re-crystallisation of salt minerals lead to granular disintegration of rocks (Alexandrowicz and Pawlikowski 1982; Rodriguez-Navarro and Doehne 1999; Słaby et al. 2000; Sabbioni 2003; Rembiś and Smoleńska 2010).

The sulphates result from the presence of SO_2_, a common component of air pollutants. Its two-pronged deposition, wet one (periodic effect of, e.g. acid rains) and so-called dry one (long-term impact of SO_2_ on the surface of stones, where sulphuric acid immediately forms in the reaction with the moisture present in the rock), is the cause of gypsum precipitation (Manecki et al. 1997; Winkler 1997; Labus and Bochen 2012). The sources of chloride ions may be represented by air pollutants and chemicals used in removing ice from streets and pavements (chiefly NaCl and CaCl_2_). An impact of chloride aerosols brought by winds from the nearby Vistula River, polluted by salty mine waters from the Upper Silesian Coal Basin, was also possible in previous decades. However, discharges of such waters have been considerably limited at present.

Calcium, barium and sodium ions originate from the decomposition of primary aluminosilicates (calcium also from dissolution of carbonates) of the sandstones. Nevertheless, significant amounts of these elements may also be linked with particulate air pollutants (Sabbioni and Zappia 1992; Marszałek 2000; Jabłońska et al. 2001; Wilczyńska-Michalik 2004; Toniolo et al. 2006) and capillary transport from the ground. With regard to the sodium ion in halite (NaCl) present in the sandstone samples collected low above the pavement level (the plinths at the Ethnographic Museum and the Czartoryski Museum), the latter process is probably of significant importance. Nevertheless, the most indicative of the effects of anthropogenic pollution are the spherical particles of aluminosilicate glass and iron oxides observed on the stone surface.

### Chemical composition of the crusts

**Fig. 9:**
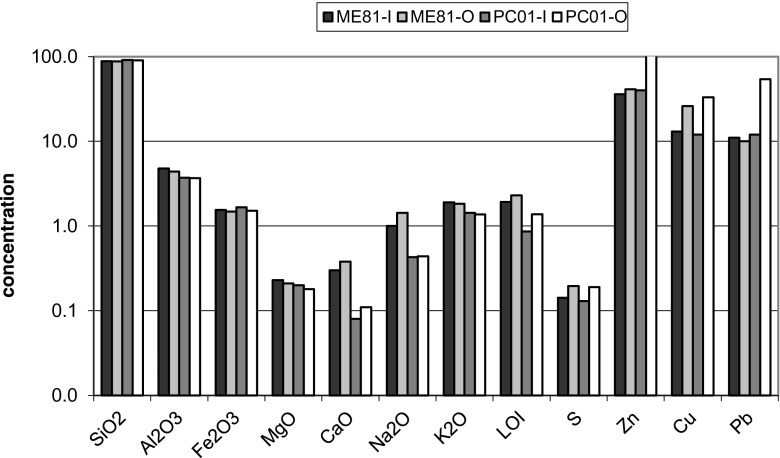
Bulk chemical composition (combined ICP-OES, ICP-MS and INAA analyses) of inner and outer layers of the sandstones crusts from architectonic details (major elements and sulphur in wt%, trace metals in ppm). *ME-81* the Ethnographic Museum, *PC-01* the Czapski Palace, *I* inner layer, *O* outer layer

Comparing the chemical compositions of crusts on the sandstones from natural outcrops and those from architectonic elements, higher concentrations of MgO, CaO, Na_2_O, S, Cu and Zn were found in the latter (Table 2). The samples represent outermost layers, ca. 0.5-cm thick, and may contain a certain admixture of a material from below as it was not always possible to separate the crust layer alone. In the case of samples collected in Kraków, these results do not differ considerably from those of the layers situated deeper, ca. 2 cm below the crust surface, which probably reflects advanced changes reaching deep into the sandstones. Nevertheless, in the inner layers of the sandstones from the architectonic elements the assays for CaO, Na_2_O and losses on ignition (LOI), and also the contents of sulphur and some trace elements (Zn, Cu) slightly decrease, whereas the contents of SiO_2_, Al_2_O_3_, Fe_2_O_3_, MgO and K_2_O slightly increase in comparison with the outer layer (Fig. 9). Such a trend manifests the accumulation of secondary salt minerals (gypsum and halite) as well as particulate pollutants (the latter are carriers of heavy metals) on sandstone surfaces and their lower amounts in subsurface layers. Similar tendencies were described, e.g. by Thomachot and Jeannette (2004), Wilczyńska-Michalik (2004) and Kamh (2005).

**Table 2:** Major (wt%) and some trace (ppm) element concentrations (combined ICP-OAS, ICP-MS and INAA analyses) in the weathering crust of the sandstones (KB-10, SMC-11, SMC-13, SMC-14—samples from Carpathian Foothill; ME-81—the Ethnographic Museum, PC-01—the Czapski Palace)

	KB-10	SMC-11	SMC-13	SMC-14	ME-81	PC-01
SiO_2_	87.83	90.60	89.61	86.64	87.83	90.10
TiO_2_	0.15	0.27	0.26	0.27	0.01	0.06
Al_2_O_3_	4.09	4.28	4.18	4.47	4.39	3.67
Fe_2_O_3_	3.40	1.08	1.42	3.49	1.47	1.50
MnO	0.01	0.01	0.01	0.01	0.01	0.01
MgO	0.09	0.08	0.08	0.08	0.21	0.18
CaO	0.06	0.08	0.09	0.19	0.38	0.11
Na_2_O	0.37	0.33	0.33	0.39	1.43	0.44
K_2_O	1.97	1.83	1.79	1.90	1.83	1.37
P_2_O_5_	0.15	0.05	0.80	0.15	0.276	0.248
LOI	1.34	1.15	1.28	2.18	2.30	1.38
Sum	99.46	99.76	99.85	99.77	100.67	99.95
S (wt%)	0.024	0.015	0.042	0.038	0.195	0.190
Cu (ppm)	9	11	15	10	26	33
Zn (ppm)	34	10	16	26	41	154
Sr (ppm)	38	35	36	43	45	36
Ba (ppm)	406	356	353	437	289	261
Pb (ppm)	20	15	20	30	10	54

*LOI* loss on ignition

In natural outcrops, spatial differences of the chemical composition between the weathering crust and an inner sandstone layer are associated, first of all, with the accumulation of iron compounds and the prevailing type of Fe-bearing minerals (Rzepa et al. 2011; Alexandrowicz et al. 2014).

The phase and chemical composition of weathering crusts indicate that their development is controlled by the environmental conditions combined, in particular, with climate. The Małopolska Voivodeship, in which the study region is situated, is one of the most polluted areas in Poland (Wilczyńska-Michalik 2004; Pająk 2011), and the Kraków conurbation is characterised by very high levels of gaseous and particulate pollution. They inherently result from adverse atmospheric conditions of Kraków, situated in the Vistula River valley surrounded by hills, as well as from a proximity of large industrial centres, and further aggravated by distant industrial emissions. The annual average concentrations of total dust particulate matter <10 μm (PM10) particles, nitrogen dioxide and sulphur dioxide recorded in a measuring station situated in the city centre are high. As regards PM10 and NO_x_, their concentrations markedly exceed permissible standards (Table 3). The excessive concentrations of NO_x_, SO_2_ and PM10 are recorded most often during the heating season, which usually lasts from October till March. Local boiler houses and household furnaces that use solid fuels (coal and/or coke) of variable (often inferior) quality are additional sources of pollution. Road transport also contributes its share, quite significant in case of any old town with its dense, structural layout.

**Table 3:** Average SO_2_, NO_2_ and PM10 concentrations (μg/m^3^) in the air in Kraków and Ciężkowice (Carpathian Foothill) in 2010

City	Average concentrations (μg/m^3^)								
	SO_2_			NO_2_			PM10		
	Annual	Summer season	Heating season	Annual	Summer season	Heating season	Annual	Summer season	Heating season
Kraków	11	4.5	18	70	71	70.5	79	48.6	91.8
City Centre, I. Krasińskiego Ave.									
Ciężkowice	8.5	3.8	13.1	10.8	8.9	12.7	45	n.m.	n.m.
Carpathian Foothill									
Permissible standards	20	–	–	40	–	–	40	–	–

From Ogar et al. (2011) and Małopolska air monitoring (2014)

*n.m.* not marked; summer season 1.IV.-30.IX., heating season 1.X.-31.III.

The area of the Carpathian Foothill (the region where the samples from the natural outcrops were collected) is affected by industrial emissions from Kraków and neighbouring towns such as Tarnów and Nowy Sącz (Lach and Wilczyńska-Michalik 1996; Gabała and Kühne 2002; Gołębiowska et al. 2002; Karnia et al. 2007; Ogar et al. 2011). The state of its atmosphere is worsened by local pollution sources associated with industrial activities, combustion of fuels (during heating season) and road transport. Nevertheless, the overall air quality is much better there than in the Kraków urban agglomeration. The annual average concentrations of sulphur dioxide, nitrogen oxides and PM10 suspended dust stay on much lower levels than those within Kraków (Table 3). In the study area, the load of acidifying compounds introduced via atmospheric precipitation: SO_4_^−2^ > N > Ca > Cl^−^ > Na > K > Mg > Zn > P > Fe > Cu > Mn > H^+^ > Pb > Ni > Cd > Cr is high, although the last decade shows a declining trend. The deposition of sulphate and nitrate ions in the Małopolska region is the highest in Poland (Pająk 2011).

The different level of the environmental pollution in the two subareas studied is reflected in the concentrations of salts, size of their crystals and the state of preservation of sandstone surfaces. Gypsum, which fairly sporadically occurs on the rock surfaces in natural outcrops, develops into tabular, relatively large and well-formed crystals (Fig. 6a). The crystals of this sulphate found on the architectonic details of historic buildings in Kraków are, as a rule, finer and lath-shaped, and occur in aggregates densely covering the surface (Fig. 6c). Such differentiation of the size and morphology probably reflects the conditions prevailing during precipitation: high concentrations of acid air pollutants (including SO_2_) result in gypsum crystallising from more saturated solutions, thereby producing a higher number of relatively small crystals (Maurice 2009). On the surfaces of the tors in the Carpathian Foothill, crystallisation is slower and proceeds from less concentrated solutions, which results in the formation of large, euhedral gypsum crystals. Nevertheless, the airborne particles observed there (aluminosilicate glass and the spherical forms of iron oxides) provide an evidence of a far-reaching industrial pollution. Thus, this contamination must be a reason of the sulphate salts and chlorides precipitating on the surfaces of the sandstone tors. It seems, however, that the main source of the cations (K, Ca, Na, Ba and Fe) necessary to the formation of these salts must rather be contributed to the weathering of mineral rock components.

There is a certain difference in the mineral composition of weathering crusts from both subareas studied: those developed on the sandstone architectonic elements contain only gypsum and halite, whereas those from the sandstone in natural exposures in addition to gypsum and halite also traces of other mineral phases: alum, barite or jarosite. The most probable explanation is that these minor salt phases have other cations than the Na^+^ and Ca^2+^ composing gypsum and halite. The additional cations must have been released in the natural environment during weathering of aluminosilicates, for instance, potassium from micas and feldspars. In an urban environment, the anthropogenic supply of sodium and calcium is so high that the two cations dominate porous solutions in sandstones. Another factor must also be taken into considerations: the sulphates present in the crusts from natural exposures crystallise under distinctly acid conditions. Jarosite is formed at very low pH values (Bingham and Nordstrom 2000), and barite also favours acid conditions. In turn, the Al-containing sulphates precipitate when aluminium is released into weathering solutions (Navrátil et al. 2013). This element is mobile when the solutions are either strongly acidic or strongly basic; otherwise, it tends to precipitate (Fest et al. 2007). Urban environments have high concentrations of dusts, most of which are alkaline and neutralise the acid reaction of airborne deposits. In the distant, natural environments of the tors, the amounts of the industrial particulates are distinctly lower and, as a result, insufficient to neutralise acid depositions and to prevent other processes, e.g. weathering of pyrite, which leads to the formation of sulphuric acid. Another acidifying factor may be represented by organic acids produced by moss and lichens colonizing the surfaces of natural exposures of sandstones. The factors mentioned combined with a low acid neutralisation capacity of waters developed within the sandstones of a low calcium content (Table 2) result in an acid reaction of the porous waters (Navrátil et al. 2013), which favours crystallisation of the sulphates mentioned above.

It should also be emphasised that the sandstones forming the tors occur in their natural geological environment. On the other hand, the sandstones used as building stones are purposely selected rock materials, additionally modified in stonework operations. Therefore, in contrast to those in the natural outcrops, they do not have immediate contact with the parental substrate, and stone blocks are often arranged in various positions and additionally cemented with calcareous or clayey material. Therefore, masonry may also affect future weathering processes. Water circulation, essential in weathering in the tors, proceeds both upwards and downwards, is usually limited in building stones, which intensifies evaporation and precipitation of secondary salts. The recurrent precipitation stress acting along the borders of external (affected) and internal (less affected or not affected at all) parts of any rock, particularly of a building stone, is a main factor of its damaging by exfoliation.

In sandstones that form natural outcrops, the content of salt minerals is not high. They facilitate hardening of rock surfaces via silification and cementation with iron compounds and protect the rock from damage exerted by atmospheric factors. Despite of this, with the passage of time, the stress caused by the differences in the coefficients of thermal expansion, in moisture contents between the rock surface and its inner parts, and in the composition of rock cement, result in natural loosening of the rock and exfoliation of weathering crust fragments. In the process, the crust substrate is exposed, which—in a further course of chemical weathering—gradually transforms and develops a new protective crust layer.

## Conclusions

The weathering crusts developed on the Carpathian sandstones exposed to various conditions of air pollution show differences in their development. The black crusts collected in highly (Kraków) and low-polluted (Carpathian Foothill) areas differ in both their fabric and composition. The sandstone black crust from tors in the Carpathian Foothill is rich in organic matter and composed of amorphous, opal-type silica. Sulphate incrustations (mainly gypsum) accompanied by spherical iron oxide or aluminosilicate glass particles from power plant emissions have been sometimes observed. In trace amounts can also appear other phases: alum, barite or jarosite. The black crust is underlain by a zone coloured by iron compounds (goethite and/or hematite). The enrichment of the surface crust in silica and iron (oxyhydr)oxides hardens its surface and lowers its porosity, thus protecting the rock interior from atmospheric agents. In contrast, the sandstones from architectonic details in monuments are loosened and covered in places by a thin black or dark grey crust, enriched in organic matter. Spherical particles of aluminosilicate glass and iron oxides (hematite and magnetite) from industrial emissions are common here. On the rock surface and also in intergranular spaces, numerous salts crystallise, mainly gypsum and halite. In contrary to the weathering crust developed on natural sandstone outcrops, these occurring on monuments enhance deterioration of the rock and contribute to mechanical disintegration of the stone. Different levels of environmental pollution in the two subareas studied are also reflected in the concentrations and size of salt crystals. Gypsum, sporadically occurring on the rock surfaces in natural outcrops, develops into large and well-formed crystals, because the precipitation of this is slower and proceeds from less concentrated solutions. The crystals found on the architectonic details are finer and densely cover the surface, because high concentrations of acid air pollutants result in gypsum crystallising from more saturated solutions.

The mechanism of developing weathering crust is similar on the surfaces of sandstone tors and of architectonic sandstone elements. The course of weathering of rock surfaces depends besides inherent petrographic features (mineral composition, texture, structure) also upon environmental conditions, being particularly affected by atmospheric pollution. In the regions of low pollution, the sandstone surfaces become covered with the natural weathering crust that provides a long-term protection of inner rock layers from further alterations. The weathering crust developed as an effect of pollution in urban areas contains considerable amounts of secondary salt minerals, mainly gypsum, reduces the resistance of surface stone layers to weathering and, as a side effect, lowers the stone aesthetic quality. The crust layer additionally sealed with airborne particulates hampers a free exchange of stone moisture with the air, a migration of soluble salts from inner parts of stones outwards and their crystallisation on the surface. All this leads to the loosening of the rock structure and finally, to the granular disintegration of the stone.

The difference in the type of salt minerals found in weathering crusts formed on the Carpathian sandstones used as architectonic elements (only gypsum and halite) and those occurring in natural outcrops (where gypsum and halite are occasionally accompanied by alum, barite or jarosite) may also result from various amounts of airborne dusts and local colonisation of natural rock exposures by moss and lichens. These factors affect the reaction of solutions migrating within the rocks, and due to that change, the composition of salt precipitates.

A certain impact on the process of weathering can also be exerted by differences in water circulation in the sandstones remaining in their natural (geological) environment and those used as building materials in towns. The sandstones of natural outcrops are characterised by easier circulation of capillary or atmospheric waters. In contrast, a block of building stone shows limited, usually one-directional water circulation with the prevalence of surface moisturising and rapid evaporation. As a consequence, the growth of weathering crusts on tors is slower than that on weathering sandstones of urban buildings.
